# Efficacy and safety of 9 nonoperative regimens for the treatment of spinal cord injury: A network meta-analysis

**DOI:** 10.1097/MD.0000000000008679

**Published:** 2017-11-27

**Authors:** Da-Nian Ma, Xia-Qi Zhang, Jie Ying, Zhong-Jun Chen, Li-Xin Li

**Affiliations:** a Department of Orthopedics; b Department of Clinical Research Center, Xuyi People's Hospital, Huaian; c Department of Neurosurgery, First Affiliated Hospital of Nanjing Medical University, Nanjing, P.R. China.

**Keywords:** clinical controlled trials, efficacy, network meta-analysis, nonoperative regimen, safety, spinal cord injury

## Abstract

**Objective:**

This network meta-analysis aims to compare the efficacy and safety of 9 nonoperative regimens (placebo, pregabalin, GM-1 ganglioside, venlafaxine extended-release [venlafaxine XR], fampridine, conventional over-ground training [OT], body-weight-supported treadmill training [BWSTT], robotic-assisted gait training [RAGT] + OT and body-weight-supported over-ground training [BWSOT]) in treating spinal cord injury (SCI).

**Methods:**

Clinical controlled trials of 9 nonoperative regimens for SCI were retrieved in the electronic database. Traditional pairwise and Bayesian network meta-analyses were performed to compare the efficacy and safety of 9 nonoperative regimens for the treatment of SCI. Weighted mean difference (WMD), odds ratios (OR), and surface under the cumulative ranking curve (SUCRA) were calculated using the Markov Chain Monte Carlo engine Open BUGS (V.3.4.0) and R (V.3.2.1) package gemtc (V.0.6).

**Results:**

A total of 9 clinical controlled trials meeting the inclusion criteria were selected in this meta-analysis. On the aspect of efficacy, the results of pairwise meta-analysis indicated that the RAGT + OT and BWSOT might have the best efficacy in SCI patients in terms of a lower extremity motor score (LEMS) compared with conventional OT; the efficacy of RAGT + OT on SCI patients was relatively better than that of conventional OT in terms of walking index for spinal cord injury (WISCI). With the aspect of safety, the constipation rate of placebo on SCI patients was relatively higher than that of venlafaxine XR; however, with respect to headache and urinary tract infection, there was no significant difference in the safety of placebo, pregabalin, GM-1 ganglioside, venlafaxine XR, and fampridine on SCI patients. The results of SUCRA values suggested that BWSOT had the highest SUCRA value (75.25%) of LEMS; RAGT + OT had the highest SUCRA value (88.50%) of WISCI; venlafaxine XR had the highest SUCRA value (94.00%) of constipation; venlafaxine XR had the highest SUCRA value (80.00%) of headache; GM-1 ganglioside had the highest SUCRA value (87.75%) of urinary tract infection.

**Conclusion:**

Our results provide evidence that the RAGT + OT and BWSOT might have the best efficacy in the treatment of SCI, and the venlafaxine XR and GM-1 ganglioside showed adequate safety for SCI.

## Abstract 2

Supplemental Digital Content is available in the text

## 1 Introduction

Spinal cord injury (SCI) is a devastating condition that frequently with sudden loss of motor, sensory, and autonomic function which is distal to the level of trauma.^[1]^ The prevalence of acute and chronic SCI in the United States exceeds 10,000 per year, leading to 720 cases per million persons bearing permanent disability each year.^[2]^ The causes of SCI include penetrating nonpenetrating lesions from vehicular accidents (38%), bullet wounds and other forms of violence (26%), sports accidents (7%), and falls (22%), particularly in elderly persons.^[3]^ Many pharmacologic treatment options, such as antidepressants, antispasticity medications, analgesics, and anticonvulsants, are restricted by the appearance of severe side effects, a lack of efficacy, or a lack of enough clinical trial data so as to support their use.^[4,5]^ However, the treatment of SCI still remains largely palliative: handling spasticity, preventing injury progression; dysautonomia, and deafferentation pain syndromes; managing complications of sensory loss; carrying out bowel and bladder training regimens; as well as teaching patients to deal with their disabilities.^[6]^ Thus, it is urgent to seek for new therapeutic strategies for treating SCI patients as well as to broaden our knowledge on both the cellular and molecular pathophysiology of SCI.

Pregabalin, as an alpha (2)-delta ligand which has analgesic, anxiolytic, and anticonvulsant properties, is known as the only Food and Drug Administration (FDA)-approved therapy for neuropathic pain on account of SCI in the United States.^[7]^ Gangliosides are complex acidic glycolipids that are present in central nervous system cells with high concentrations, and they form a main part of the cell membrane and are predominantly situated in the outer leaflet of the bilayer of cell membrane.^[8]^ Venlafaxine XR is a dual-action antidepressant, which has been increasingly used in clinical practice.^[9]^ To be specific, venlafaxine XR is effective in SCI patients diagnosed with major depressive disorder.^[10]^ Fampridine (also known as 4-aminopyridine) is a special blocker of voltage-dependent and neuronal potassium channels that in demyelinated axons.^[11]^ Clinical studies have revealed that oral or intravenous administration of fampridine decreased spasticity and improved motor and sensory function in patients with SCI.^[12,13]^ Robot-assisted gait training (RAGT), conducted on a driven gait orthosis, is regularly performed over a longer period in comparison with treadmill training.^[14]^ Additionally, RAGT supplies a more supportive environment together with normalized physiological gait training, which has the advantages of temporal aspects and ideal kinematics, and those patients who have severely affected hemiplegia showing better outcomes treated with RAGT than with treadmill training.^[15]^ Body-weight-supported over-ground training (BWSOT), often used in neurological rehabilitation, is an alternative to walking on a treadmill, while body-weight-supported treadmill training (BWSTT) is easier for the doctor to provide manual guidance, so as to cause less of a manual handling risk.^[16]^ Meanwhile, a previous study suggested that BWSTT could improve gait symmetry and velocity surpassing over-ground training (OT), while the results did not conclude that these 2 training methods are equally beneficial.^[17]^ However, at present, there is still a lack of comprehensive research on the actual evaluation of the efficacy and safety of 9 nonoperative regimens.

Traditional meta-analysis is used to synthesize the information of different trials assessing the same intervention(s) so as to obtain an overall estimation of the treatment effect for an intervention to the control.^[18]^ Network meta-analysis, as a generalization of pairwise meta-analysis, allows for indirect comparisons of interventions that not studied in a head-to-head fashion, and it is possible to develop a network meta-analysis where all the trials have at any rate one intervention with another in common.^[19]^ Therefore, this network meta-analysis aims to compare the efficacy and safety of 9 nonoperative regimens (placebo, pregabalin, GM-1 ganglioside, venlafaxine XR, fampridine, conventional OT, BWSTT, RAGT + OT, and BWSOT) on patients with SCI.

## 2 Materials and methods

### 2.1 Search strategy

The electronic database of PubMed and Cochrane Library (from their inceptions to December 2016) together with a manual search was retrieved for relevant references. With the combination of keywords and free words, the search words mainly included SCI, nonoperative regimens, efficacy, safety, drug therapy, clinical controlled trials, etc.

### 2.2 Inclusion and exclusion criteria

The inclusion criteria were: research type: clinical controlled trials; intervention measures: placebo, pregabalin, GM-1 ganglioside, venlafaxine XR, fampridine, conventional OT, BWSTT, RAGT + OT, and BWSOT; research subjects: patients aged 16 to 71 years old with SCI; the studies with constipation, urinary tract infection, and lower extremity motor score (LEMS) as outcome indicators. The exclusion criteria were: SCI patients with neurological and orthopedics disorders (such as head injury); patients had a history of schizophrenia or bipolar disorder; patients with clinically significant renal and liver disease or severe coronary artery disease; studies lack of integrity of the literature data (such as nonpaired study); nonclinical controlled trials; repeated published literature; meeting report, systematic review or summary article; non-English article.

### 2.3 Data extraction and quality evaluation

Two investigators independently extracted the data from the included studies using a predefined form. If there is a dispute in the process of data extraction, all investigators would discuss and reach a consensus through consultation. Clinical controlled trials were evaluated by more than 2 investigators based on Physiotherapy Evidence Database (PEDro) scale.^[20]^ The total scores of PEDro were 11 points: ≥4 points were regarded as high quality and <4 points were regarded as low quality.^[21]^ For each domain, the low, high, or unclear risk of bias was assigned by a judgment of “yes,” “no,” or “unclear,” respectively. The study was considered as low risk of bias if one or no domain was decided “unclear” or “no.” The study was regarded as moderate risk of bias if 2 or 3 domains were considered “unclear” or “no.” The study was regarded as high risk of bias if 4 or more domains were considered “unclear” or “no.”^[22]^ Quality assessment together with investigation of publication bias was performed using Review Manager 5 (RevMan 5.2.3, Cochrane Collaboration, Oxford, UK).

### 2.4 Statistical analysis

Firstly, traditional pairwise meta-analyses for enrolled studies which directly compared different treatment arms were performed. The pooled estimates of odd ratios (ORs), weighted mean difference (WMD) as well as 95% credible intervals (CrIs) were reported in our results. In order to assess the heterogeneity among the studies, Chi-square test and *I*-square test were used.^[23]^ Secondly, R 3.2.1 software was utilized to draw the network diagram. Each node stands for a kind of intervention, the node size represented the sample size, and the thickness of lines between nodes represented the number of included studies. Thirdly, Bayesian network meta-analyses were carried out to compare different interventions to each other. Based on noninformative priors, each analysis was conducted for effect sizes and precision. After a 20,000-simulation burn-in phase and 4 chains, convergence together with lack of autocorrelation were checked and confirmed. Finally, direct probability statements were taken from an additional 50,000-simulation phase.^[24]^ To providing help to the interpretation of ORs and WMDs, we calculated the probability of each intervention being the most effective or safest treatment method on the basis of a Bayesian approach using probability values which were summarized as surface under the cumulative ranking curve (SUCRA). The larger the SUCRA value is, the better the rank of the intervention would be.^[25,26]^ R (V.3.2.1) package gemtc (V.0.6), along with the Markov Chain Monte Carlo engine Open BUGS (V.3.4.0), was used for all computations.

## 3 Results

### 3.1 Baseline characteristics of included studies

**Figure 1:**
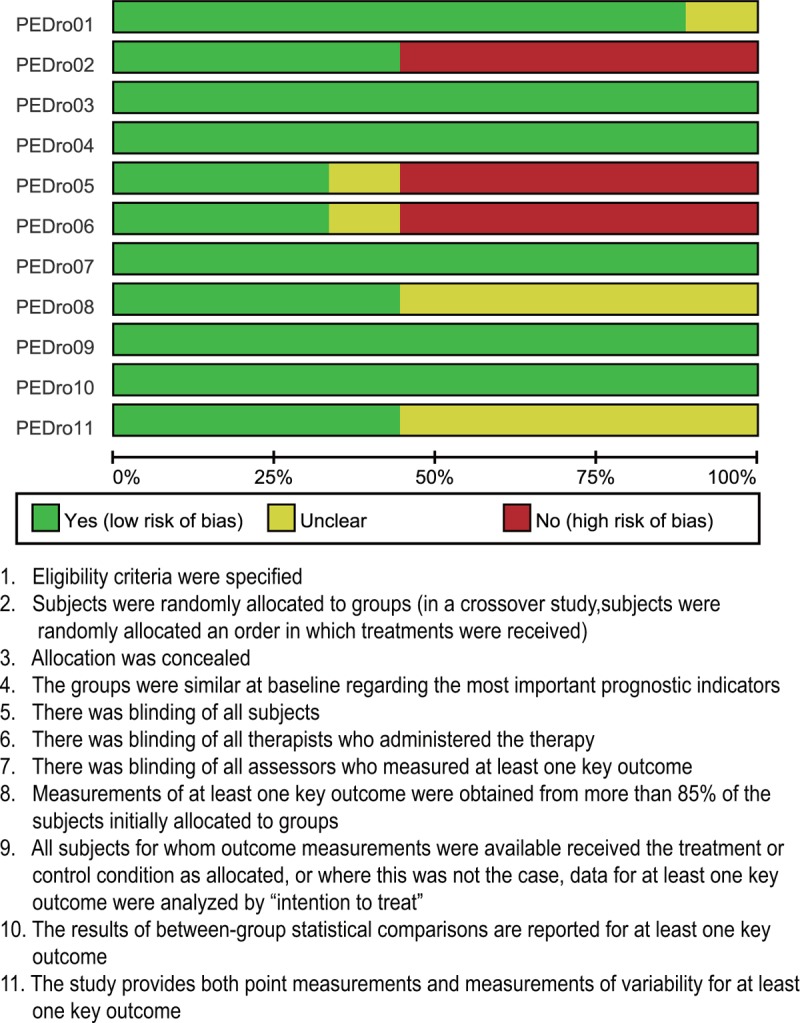
Physiotherapy Evidence Database (PEDro) scale for the quality assessment of enrolled studies.

**Figure 2:**
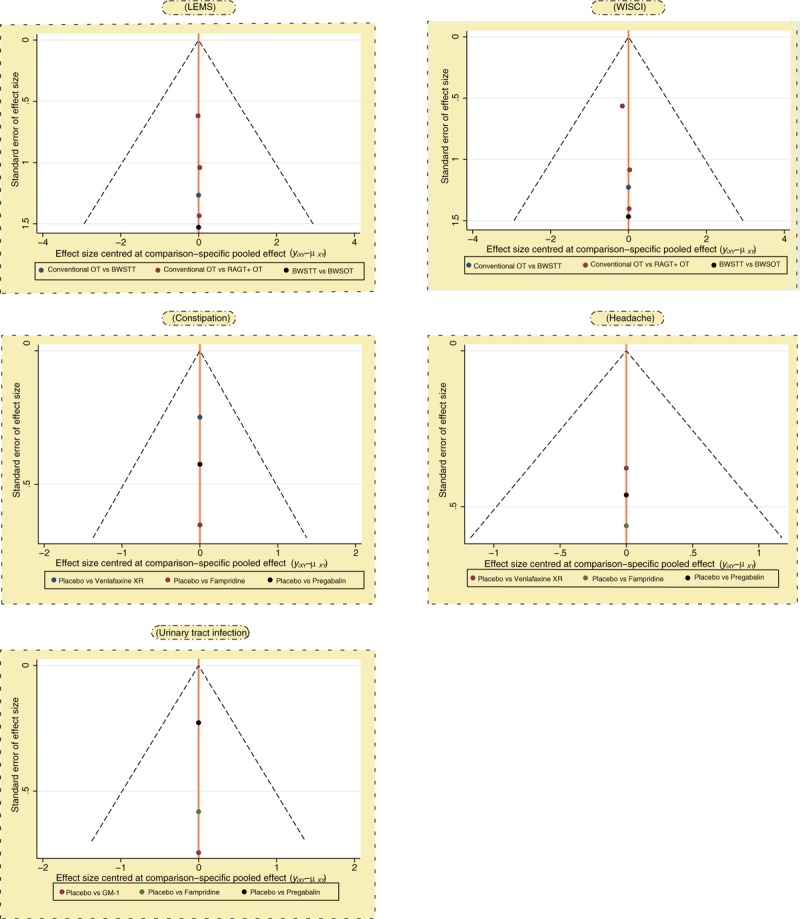
Funnel plot for publication bias of included studies. BWSOT = body-weight-supported over-ground training, BWSTT = body-weight-supported treadmill training, LEMS = lower extremity motor score, OT = over-ground training, RAGT = robotic-assisted gait training, WISCI = walking index for spinal cord injury.

According to the search strategies, 3653 relevant articles were identified. After removing 6 duplicates, 985 letters or reviews, 674 nonhuman articles, and 283 non-English articles, a total of 1705 articles were evaluated for eligibility by full-text review. After that, 1696 articles were rejected due to the follows: 866 for noncohort studies, 623 for studies unrelated to SCI, 201 for studies unrelated to nonoperative regimens, and 6 for studies without data or complete data. Finally, we identified 9 clinical controlled trials that met the inclusion criteria, in which there were a total of 841 SCI patients^[12,27–34]^ (Fig. S1). Most of these patients were treated with placebo. The publication times of those included studies were from 1991 to 2015. Among the 9 included studies, the subjects in 7 studies were from Caucasian populations, and the subjects in 2 studies were from Asian populations; all the included studies were 2-arm trials. The baseline characteristics of the included studies are shown in Table 1, and the PEDro scale for the risk of bias is shown in Fig. 1. Besides, Fig. 2 shows that scattered points are located in the funnel and are evenly distributed on both sides of the center line, which indicates that the included studies have no significant publication bias.

**Table 1:**
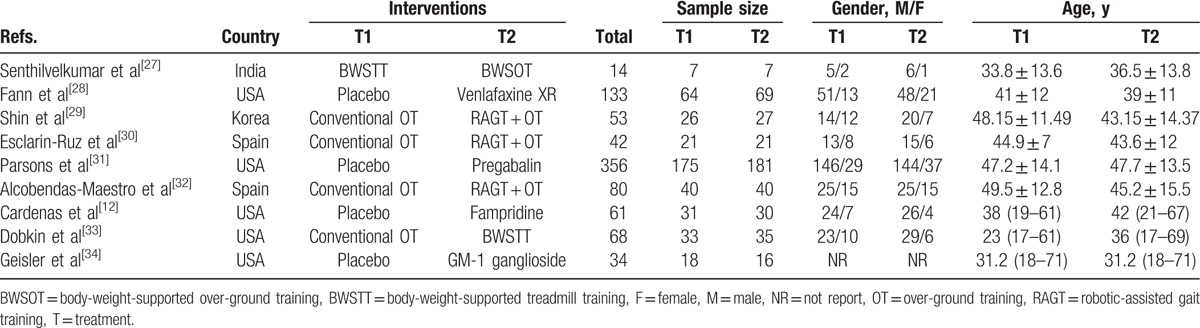
The baseline characteristics for included studies.

### 3.2 Pairwise meta-analysis

We directly compared the therapeutic efficacy and safety of these 9 nonoperative regimens on SCI. On the aspect of efficacy, conventional OT had relatively poorer efficacy on SCI patients in terms of LEMS compared with RAGT + OT and BWSTT (WMD = −2.01, 95% CI = −2.58 to −1.44; WMD = −3.00, 95% CI = −4.20 to −1.80, respectively); the efficacy of conventional OT on SCI patients was relatively poorer than that of RAGT + OT in terms of walking index for spinal cord injury (WISCI) (WMD = −4.00, 95% CI = −7.55 to −0.46) (Table 2). On the aspects of safety, the constipation rate of placebo on SCI patients was relatively higher than that of venlafaxine XR (OR = 2.70, 95% CI = 1.18–6.21). However, with respect to headache and urinary tract infection, there was no significant difference in the safety of placebo, pregabalin, GM-1 ganglioside, venlafaxine XR, and fampridine on patients with SCI (Table 3).

**Table 2:**
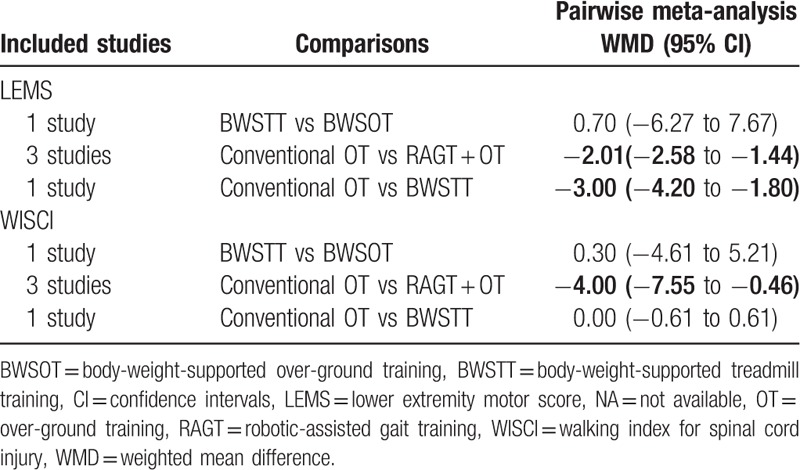
Pairwise meta-analysis of improvements in lower extremity motor score and walking index for spinal cord injury.

**Table 3:**
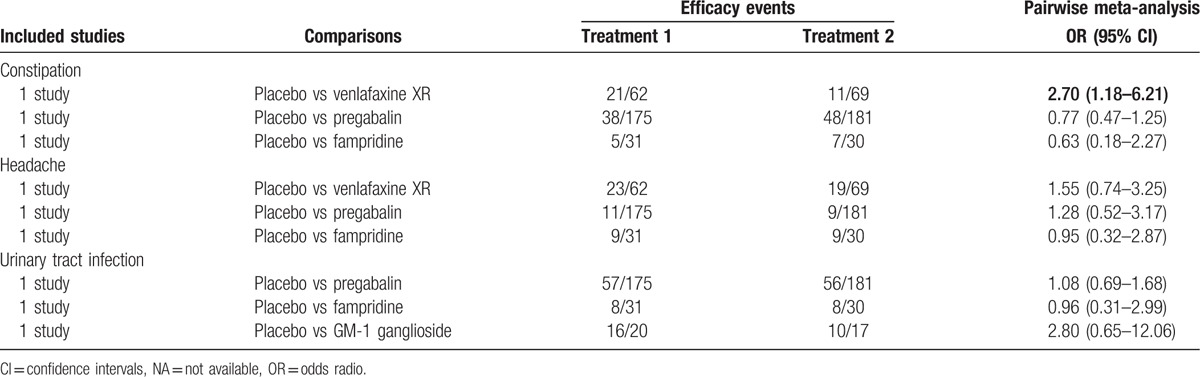
Estimated odds ratios and 95% confidence intervals from pairwise meta-analysis.

### 3.3 Network evidence

**Figure 3:**
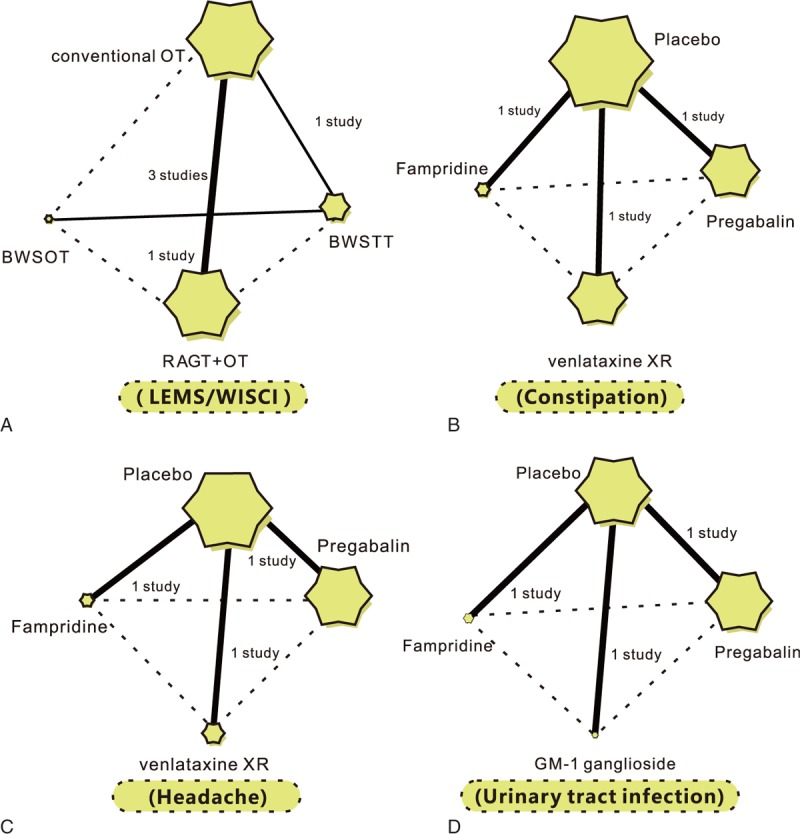
Evidence diagrams for the efficacy and safety of 9 nonoperative regimens on patients with spinal cord injury. BWSOT = body-weight-supported over-ground training, BWSTT = body-weight-supported treadmill training, LEMS = lower extremity motor score, OT = over-ground training, RAGT = robotic-assisted gait training, WISCI = walking index for spinal cord injury.

Among these regimens, we found that on the aspects of efficacy, the majority of SCI patients were treated with conventional OT in terms of LEMS and WISCI, while the number of SCI patients receiving BWSOT treatment was the least; on the aspects of safety, most of SCI patients were treated with placebo in terms of constipation, headache and urinary tract infection, while the number of SCI patients receiving GM-1 ganglioside and fampridine treatment was relatively less (Fig. 3).

### 3.4 Main results of network meta-analysis

The results of this network meta-analysis indicated that the efficacy of RAGT + OT on SCI patients was relatively better than that of conventional OT in terms of LEMS (WMD = 2.06, 95% CI = 0.08–4.09) (Table 4). However, with respect to WISCI, constipation, headache, and urinary tract infection, there was no significant difference in the efficacy and safety of placebo, pregabalin, GM-1 ganglioside, venlafaxine XR, fampridine, conventional OT, BWSTT, RAGT + OT, and BWSOT on patients with SCI (Fig. S2–3 and Tables 4 and 5).

**Table 4:**
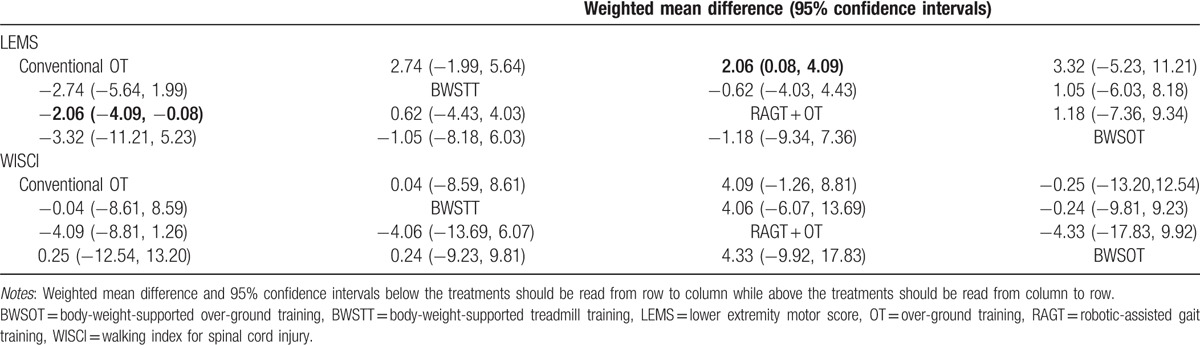
Weighted mean difference and 95% confidence intervals of 4 treatment modalities of 2 endpoint outcomes.

**Table 5:**
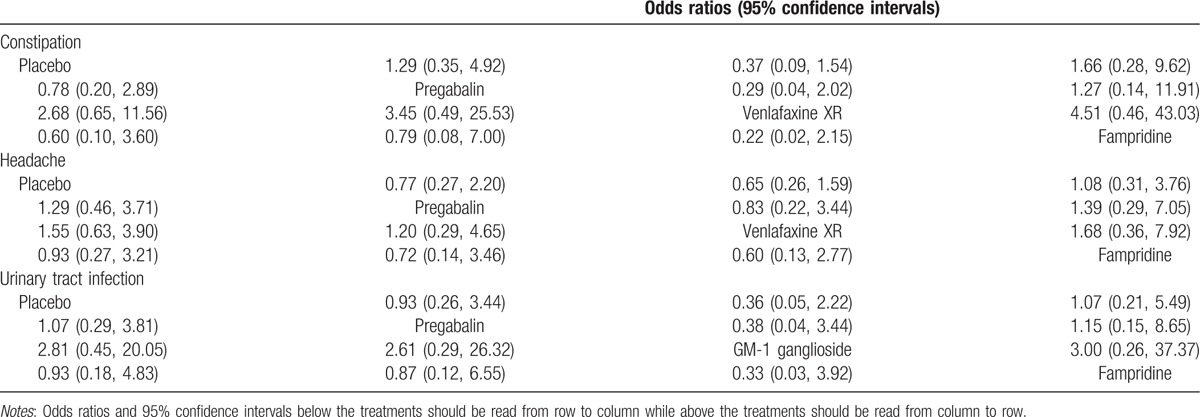
Odds ratios and 95% confidence intervals of 3 endpoint outcomes.

### 3.5 SUCRA value

**Figure 4:**
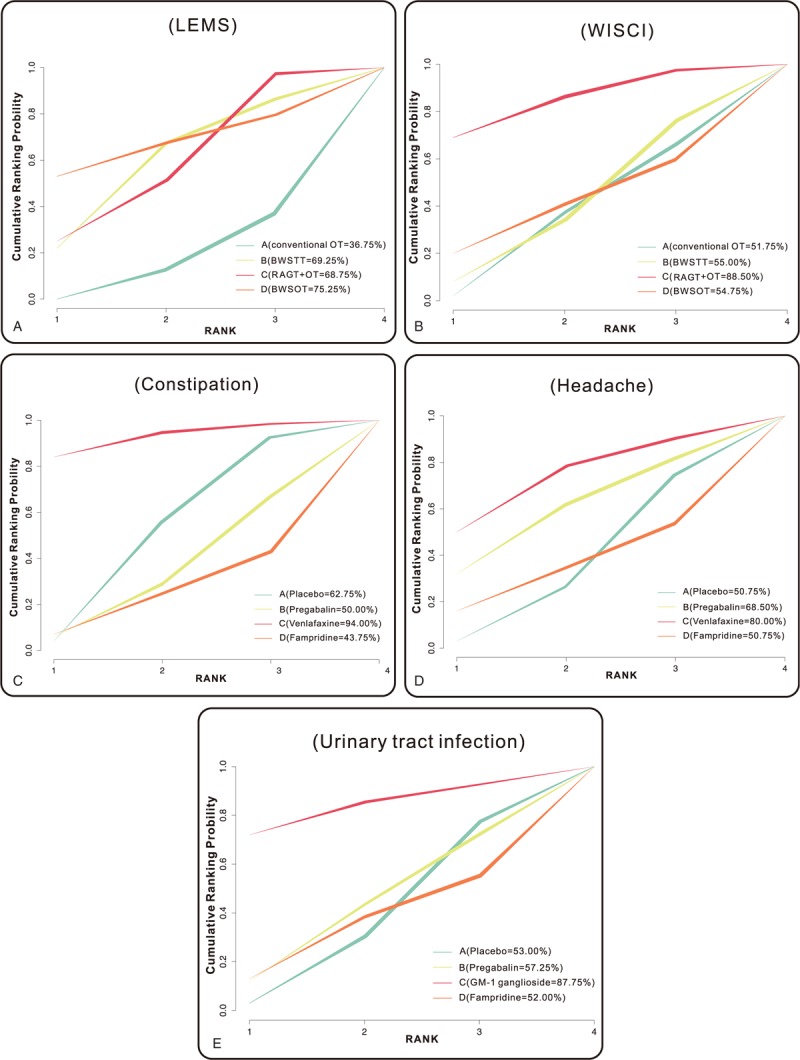
Surface under the cumulative ranking curve plots for the efficacy and safety of 9 nonoperative regimens on patients with spinal cord injury. BWSOT = body-weight-supported over-ground training, BWSTT = body-weight-supported treadmill training, LEMS = lower extremity motor score, OT = over-ground training, RAGT = robotic-assisted gait training, WISCI = walking index for spinal cord injury.

Figure 4 shows the SUCRA values of the therapeutic effects on SCI patients for the 9 nonoperative regimens. The results suggested that on the aspect of efficacy, BWSOT had the highest SUCRA value (75.25%) while conventional OT had the lowest SUCRA value (36.75%) in terms of LEMS; RAGT + OT had the highest SUCRA value (88.50%) while conventional OT had the lowest SUCRA value (51.75%) in terms of WISCI. On the aspect of safety, venlafaxine XR had the highest SUCRA value (94.00%) but fampridine had the lowest SUCRA value (43.75%) with respect to constipation; venlafaxine XR had the highest SUCRA value (80.00%), whereas, fampridine and placebo had the lowest SUCRA value (both 50.75%) with respect to headache; in terms of urinary tract infection, GM-1 ganglioside had the highest SUCRA value (87.75%) while fampridine had the lowest SUCRA value (52.00%). The results mentioned above indicated that RAGT + OT and BWSOT had better efficacies on SCI patients, while the efficacy of conventional OT was relatively poorer; venlafaxine XR and GM-1 ganglioside had better safeties on SCI patients, while the safety of fampridine was relatively poorer.

## 4 Discussion

SCI influences not only conduction of sensory and motor signals across the site of lesion, but also the autonomic nervous system.^[35]^ According to American Spinal Injury Association Impairment Scale, the degree of SCI can be divided into A—Complete, B—Incomplete, C, D—Incomplete, E—Normal.^[36]^ In this study, SCI in the included studies mainly caused by traumatic and nontraumatic injuries, such as accident, gunshot, vehicular, fall, violence. The indicators of efficacy and safety of different regimens in the included studies were measured by LEMS, WISCI, constipation rate, headache, and urinary tract infection altogether. Through the comparison of these five indicators, this study evaluated the efficacy and safety of 9 nonoperative regimens, including placebo, pregabalin, GM-1 ganglioside, venlafaxine XR, fampridine, conventional OT, BWSTT, RAGT + OT, and BWSOT, using direct pairwise meta-analysis and network meta-analysis.

The results of pairwise meta-analysis showed that the efficacies of RAGT + OT and BWSOT were relatively better, while the efficacy of conventional OT was relatively poorer, and the results of SUCRA values further confirmed this point. RAGT focused on improving the activity of spinal interneurons on the basis of the function of the central pattern generator through supplying sensory-motor stimulation to ameliorate neural plasticity.^[37]^ RAGT also has the merits of repeatedly performing a preprogrammed gait pattern, and RAGT has been reported to promote motor recovery as well as functional improvement.^[32,38]^ Meanwhile, RAGT can also improve confidence in walking performance, promote intralimb and interlimb coordination, as well as alter synergistic contraction of the antagonistic muscles of the knee joint and the ankle joint.^[39]^ However, RAGT cannot reflect an immediate feedback, and RAGT combined with conventional OT can yield more benefits in ambulatory function than conventional OT alone do, thus improving walking ability and muscle strength in SCI patients.^[29]^ For BWSOT in SCI patients, it can be a more task-specific form of gait training when compared with treadmill training, which may also relieve the transition from walking in the process of therapy to walking in a real-life environment.^[40]^ BWSOT is also appealing for the reason that it is inexpensive and also does not require costly treadmills, which is a particularly significant consideration for patients who in the low and middle-income countries.^[41]^ Moreover, BWSOT is better than conventional OT for the following aspects: it frees the doctors from physically supporting patients in the process of gait training, it decreases the need for personnel to build a safe environment for gait training, and it provides the doctors with the choice to step back, observe and further analyses the patient's gait.^[42]^

The results of pairwise meta-analysis also showed that the safeties of venlafaxine XR and GM-1 ganglioside were relatively higher, while the safety of fampridine was relatively lower, and the results of SUCRA values further confirmed this point. Venlafaxine has been tested extensively and has reported to be both safe and well tolerated.^[43]^ Additionally, venlafaxine seems to be effective for obsessive–compulsive disorder (OCD), and it causes fewer side effects than other medications.^[44]^ Compared with placebo controls, patients who treated with venlafaxine XR showed greater improvement in SCI-related disability, especially in the areas of home responsibilities or family life and social life.^[28]^ GM-1 ganglioside is an infrequent autosomal recessive lysosomal storage disorder which is attributable to a deficiency of β-galactosidase enzyme activity.^[45]^ GM-1 is often used in clinical practice and it is also described as a well-established drug in SCI medical interventions.^[46]^ The administration of GM-1 ganglioside could improve nerve functions with the mechanisms of activity reservation of Ca^2+^-ATPase and Na^+^-K^+^-ATPase; antagonism of excitatory amino acid toxicity; promotion of multiple nerve growth factors; prevention of lactic acidosis; prevention of intracellular calcium accumulation; direct restoration of damaged nerve cell membranes; blockage of nerve cell apoptosis.^[47]^ There are also studies showed an effective result of GM-1 ganglioside in treating SCI patients.^[48–50]^ However, since fampridine could significantly improve walking speed in patients with multiple sclerosis when compared with placebo, the role of fampridine as a contributory factor to the clinical efficacy was unclear,^[11]^ which is consistent with the results of our meta-analysis.

Network meta-analysis offers more advantages compared to conventional meta-analysis, and it mainly lies in the ability to the quantitative statistical analysis of different interventions for the same disease, thus selecting the optimal treatment by assessing different efficacies.^[51]^ However, our present network meta-analysis had also some limitations: there were significant differences for the number of studies on the direct comparison of various interventions and the sample size of each intervention, which might influence the overall results of the study; in this research, we cannot do the node cutting and clustering analysis due to the insufficient data of each intervention, the significant difference of the sample size of the intervention measures and a difference in the number of included studies on the direct paired comparisons among various interventions.

In conclusion, our network meta-analysis suggests that the RAGT + OT and BWSOT might have the best efficacy in the treatment of SCI, and the venlafaxine XR and GM-1 ganglioside showed adequate safety for SCI, which has a certain clinical significance for the treatment of SCI. Since there are some unavoidable limitations existed in this study, our research will focus on the appearance of new studies, particularly large sample studies in the future to strengthen our results.

## Supplementary Material

Supplemental Digital Content
