# Supplementary material for: Efficacy and safety of 9 nonoperative regimens for the treatment of spinal cord injury: A network meta-analysis
Source: Medicine (Baltimore). 2017 Nov 27;96(47):e8679. doi: 10.1097/MD.0000000000008679 (PMC5708945; doi:10.1097/MD.0000000000008679)
Supplement: Supplemental Digital Content [file medi-96-e8679-s001.doc]

**Figure S1** Flow chart for screening the included studies

**Figure S2** Relative forest plots for the efficacy and safety of nine non-operative regimens on patients with spinal cord injury in terms of lower extremity motor score and walking index for spinal cord injury (LEMS: lower extremity motor score; WISCI: walking index for spinal cord injury; BWSTT: body weight-supported treadmill training; BWSOT: body weight-supported over-ground training; OT: over-ground training; RAGT: robotic-assisted gait training)

**Figure S3** Relative forest plots for the efficacy and safety of nine non-operative regimens on patients with spinal cord injury in terms of constipation, headache and urinary tract infection
